# Comparison of two different biomaterials in the bone regeneration (15, 30 and 60 days) of critical defects in rats

**DOI:** 10.1590/ACB360605

**Published:** 2021-07-19

**Authors:** Patricia Brassolatti, Paulo Sérgio Bossini, Ana Laura Martins de Andrade, Genoveva Lourdes Flores Luna, Juliana Virginio da Silva, Luciana Almeida-Lopes, Marcos Aurélio Napolitano, Lucimar Retto da Silva de Avó, Ângela Merice de Oliveira Leal, Fernanda de Freitas Anibal

**Affiliations:** 1 PhD in Biotechnology. Postgraduate Program in Evolutionary Genetics and Molecular Biology – Department of Morphology and Pathology – Universidade Federal de São Carlos – Sao Carlos (SP), Brazil.; 2 PhD in Physiotherapy. NUPEN - Research and Education Center in Health Science and DMC Equipment Import and Export-Co. Ltda – Sao Carlos (SP), Brazil.; 3 PhD in Physiotherapy. Department of Physiotherapy – Universidade Federal de São Carlos – Sao Carlos (SP), Brazil.; 4 PhD in Biotechnology. Metabolic Endocrine Research Laboratory – Department of Medicine – Universidade Federal University de São Carlos – Sao Carlos (SP), Brazil.; 5 Graduate student in Biotechnology. Institute of Physics of Sao Carlos– Universidade de São Paulo – Sao Carlos (SP), Brazil.; 6 PhD in Science and Materials Engineering. NUPEN - Research and Education Center in Health Science and DMC Equipment Import and Export-Co. Ltda – Sao Carlos (SP), Brazil.; 7 PhD in Chemistry. DMC Equipment Import and Export–Co. Ltda – Sao Carlos (SP), Brazil.; 8 Associate Professor. Department of Medicine – Universidade Federal de São Carlos – Sao Carlos (SP), Brazil.; 9 Associate Professor. Department of Medicine – Universidade Federal de São Carlos – Sao Carlos (SP), Brazil.; 10 Associate Professor. Department of Morphology and Pathology – Universidade Federal de São Carlos – Sao Carlos (SP), Brazil.

**Keywords:** Biocompatible Materials, Skull, Bone Regeneration, Rats

## Abstract

**Purpose:**

To evaluate and compare two types of different *scaffolds* in critical bone defects in rats.

**Methods:**

Seventy male Wistar rats (280 ± 20 grams) divided into three groups: control group (CG), untreated animals; biomaterial group 1 (BG1), animals that received the *scaffold* implanted hydroxyapatite (HA)/poly(lactic-co-glycolic) acid (PLGA); and biomaterial group 2 (BG2), animals that received the *scaffolds* HA/PLGA/Bleed. The critical bone defect was induced in the medial region of the skull calotte with the aid of an 8-mm-diameter trephine drill. The biomaterial was implanted in the form of 1.5 mm thick *scaffolds*, and samples were collected after 15, 30 and 60 days. Non-parametric Mann-Whitney test was used, with the significance level of 5% (p ≤ 0.05).

**Results:**

Histology revealed morphological and structural differences of the neoformed tissue between the experimental groups. Collagen-1 (Col-1) findings are consistent with the histological ones, in which BG2 presented the highest amount of fibers in its tissue matrix in all evaluated periods. In contrast, the results of receptor activator of nuclear factor kappa-Β ligand (Rank-L) immunoexpression were higher in BG2 in the periods of 30 and 60 days, indicating an increase of the degradation of the biomaterial and the remodeling activity of the bone.

**Conclusions:**

The properties of the HA/PLGA/Bleed *scaffold* were superior when compared to the *scaffold* composed only by HA/PLGA.

## Introduction

Bone defects are often referred to as clinical problems, with high rates of morbidity and mortality^1^. The main occurrences of bone fractures are trauma result, tumor resection, congenital malformation, or degenerative diseases^2^-^4^. The consolidation of bone fracture is a complex biological process involving the spatial and temporal interaction of different cell types. It begins withthe development of the blood clot, which results from theactivation of the plasma coagulation cascade, and from this the other phases come, such as inflammation, ossification and remodeling^5^,^6^. Generally, bone tissue has high regenerative capacity, but, when it comes to areas with considerable extensions, this capacity is compromised, resulting in delayed consolidation^5^,^7^,^8^.

Although there are evidences that autogenous bone grafts may represent gold standard treatment in the medical field, they are currently considered clinically limiting because of low availability and donor-area related morbidity^9^-^11^. Therefore, tissue engineering with biotechnology stands for presenting innovative approaches to treatment through the development of composite biomaterials capable of interacting with the injury environment and assisting their recovery in a quick and safe manner^12^.

Composite biomaterial purposes the union of properties of two or more materials, with the conciliar perspective at the end of the process several properties in a new material, which in turn will possess superior biological capacity of those observed in their individual constituents. These biomaterials are generally constructed in the *scaffold* format, since they have an ideal tridimensional (3D) structure to guide cell adhesion and proliferation and can serve as conductors or reservoirs of water, nutrients, cytokines and/or growth factors^11^,^13^-^15^.

Among the materials indicated for this purpose, hydroxyapatite (HA) is one of the most used, because it is a mineral composed mainly of calcium and phosphate, with biocompatibility and osteoconductivity properties that mimic the mineral structure of the natural bone. In-vitro studies have shown that the use of HA nanocomposites helps the proliferation and differentiation of osteoblasts^16^, but they are devoid of mechanical stability^17^. Due to this, the incorporation of synthetic polymers capable of supplying such needs is sought.

Polymers considered biodegradable and synthetic as poly(glycolic) acid (PGA), polylactic acid (PLA), polycaprolactone (PCL), as well as their copolymers, are also widely used in the development of clinically acceptable *scaffolds* 18-20. It is believed that poly(lactic-co-glycolic) acid (PLGA) is an excellent polymer, because it has biocompatibility, biodegradability, water-soluble properties^20^,^21^ and the ideal mechanical properties that contribute to the stability of the compound, but rapid degradation. Thus, the association of the properties of HA with PLGA becomes interesting from the biological point of view, since its complementarity assiststhe cellular adhesion processes^22^,^23^, and also serves as the guiding vehicle for other substances or other types of material^23^. In addition, polysaccharides of vegetable origin, such as carboxymethyl cellulose and starch, can be used as strategies to act on hemostasis and coagulation of mammalian tissues. This type of natural biomaterial can be incorporated into *scaffolds* and effectively contribute to the decrease and/or control of local blood leakage by immediately activating the coagulation factors, which therefore favors the surgery itself, as well as the process of regeneration and/or repair that will occur later^24^.

Considering that composite materials based on HA/PLGA are already well indicated as potential orthopedic implants^18^, since their contribution is well established in the phases of cell proliferation and remodeling, with the intention of increasing the biological capacity of HA/PLGA and also to develop a new type of composite material, a polysaccharide with hemostatic properties named by DMC company as Bleed was added to the HA/PLGA structure, since the union of the three components can provide a more promising biological effect.

Thus, the objective of this study was to evaluate the behavior of two distinct types of biomaterial (HA/PLGA and HA/PLGA/Bleed) in bone regeneration process, mainly directed to tissue morphological aspects in critical bone defects induced in the rats calvaria.

## Methods

Animal studies were carried out after approval by the Institutional Committee on Ethics in Animal Use of the Universidade Federal de São Carlos (CEUA-UFSCar) (approval No. 051/2014).

Seventy-two male Wistar rats (*Rattus norvegicus*, var. *Albinus*, Rodentia, Mammalia) were used at three months of age and had mean body mass of 280 ± 20 grams. Animals were provided by the Central Animal Facility of UFSCar, were kept in the experimental room at the Physiotherapy Department (UFSCar), in individual polypropylene cages, in a hygienic environment with controlled temperature at 18-21°C, light-dark cycles from 12 h-12 h, and free access to commercial-type feed and water.

### Operating technique

The animals were weighed, anesthetized intraperitoneally and previously trichotomized. After the process of asepsis of the area, an incision was made in the medial region of the skullcap, in the anteroposterior direction, of approximately 1.5 cm, thus establishing the bone defect of critical size. For the induction of the lesion, a 2-cm long and 8-mm external diameter trefoil type dental drill (WMA), driven by a BELTEC (Araraquara, SP, Brazil) micromotor, with rotation of 13.500 rpm, irrigated with saline solution, was used, to avoid bone tissue burning. The drill was positioned perpendicularly to the bone surface, in order to break the external and internal cortices until the dura mater exposure, promoting a hole of 8-mm diameter. After the procedure, the suture was performed, and the animals received dipirone-sodium in 6.2 mg.kg^-1^ proportion.

### Experimental design

The animals were randomly distributed in three experimental groups (with eight animals each), and divided in three groups and three subgroups, as demonstrated in Table 1:

**Table 1:** Description of the experimental groups with the respective number of animals in each evaluated period.

Experimental groups			
Trial period	Control group	Biomaterial group 1	Biomaterial group 2
15 days	8 animals	8 animals	8 animals
30 days	8 animals	8 animals	8 animals
60 days	8 animals	8 animals	8 animals

- Control group (CG): the animals were induced to the bone defect of critical size and did not receive any type of treatment;
- Biomaterial group 1 (BG1): the animals were submitted to the bone defects and received the *scaffold* implanted composed of the HA/PLGA;
- Biomaterial group 2 (BG2): the animals were submitted to a bone defect and received the *scaffolds* implanted composed of the HA/PLGA/Bleed.

### Treatment

#### Preparation of composite HA/PLGA and HA/PLGA/Bleed

**Figure 1:**
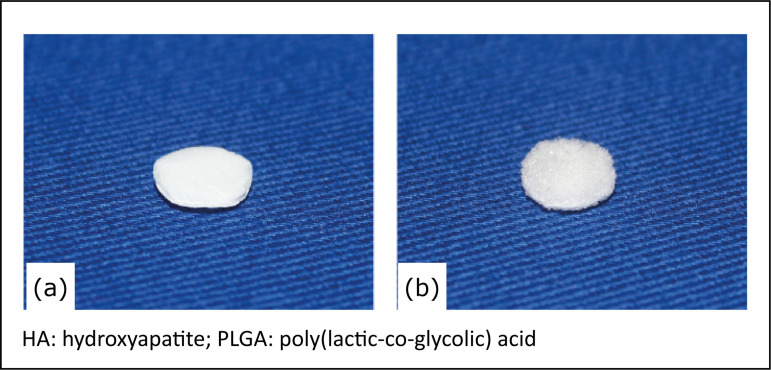
*Scaffolds* of the biomaterials used in this study. **(a)** HA/PLGA *scaffold*; **(b)** HA/PLGA/Bleed *scaffold*. The *scaffolds* of both experimental groups had 8 mm of diameter and were 1.5 mm thick.

To form the HA/PLGA composition, the commercial PLGA polymer was first dissolved in chloroform and placed in an ultrasonic bath. Next, the HA nanoparticles obtained by the calcium hydroxide precipitation method, Ca(OH)_2_ with orthophosphoric acid H_3_PO_4_, were dispersed in this bath step by step. After 10 minutes, the mixture was placed in glass plates and allowed to evaporate in an oven at room temperature for 24 hours and then transferred to a vacuum chamber for an additional 48 hours. At the end, the *scaffold* showed proportion of 30% HA + 70% PLGA with 1.5-mm thickness and 8 mm in diameter (Fig. 1a).

To obtain the new composite biomaterial (HA/PLGA/Bleed), the first blend (HA/PLGA) was used following the procedure already described. After obtaining the HA/PLGA compound, it was crushed in a knife mill and sieved in analytical sieve with known granulometry, to obtain the granules. Soon afterwards, the vegetable polysaccharide paste (Bleed) (developed and manufactured exclusively by the DMC Equipments Import and Export-Co.) was added to this initial mixture. The final suspension was lyophilized, and the *scaffold* showed proportion of 2.4% HA + 5.6% PLGA + 92% Bleed, with 1.5-mm thickness and 8-mm diameter. It should be noted that this new biomaterial composite is in the process of patent. So, there is still a business secrecy about it (Fig. 1b).

#### Euthanasia of animals and collection of samples

Euthanasia was performed by anesthetic overdose (ketamine and xylazine) at the 15th, 30th and 60th postoperative day, according to each experimental subgroup. Immediately thereafter, the region of the critical-size bone defect area was removed and sent to the processing slides needed for further analysis.

### Analysis

#### Histopathological analysis

For the histopathological analysis, the area of the critical-sized defect was fixed in 10% buffered formalin (Merck, Darmstadt, Germany) for 24 hours, decalcified in 4% EDTA solution (Merck, Darmstadt, Germany) and subsequently included in paraffin. Then, the blocks were cut in longitudinal orientation, with a standard thickness of 5 µm and mounted on histological slides. The qualitative analysis of the region of the bone defect was performed with hematoxylin and eosin (HE) stained slides (Merck, Darmstadt, Germany). For this, a light microscope (Olympus Optical Co., Tokyo, Japan) was used, in which tissue changes were observed, such as the presence of neoformed bone tissue, inflammatory process and/or granulation tissue and fibrosis.

#### Morphometric assessment

For morphometry, the slides were stained with Masson™ trichrome. For the analysis, three fields of the critical size bone defect region were selected. To measure the area of neoformed bone, a Motic Images Plus analysis system version 2.0 was used. The areas in turn were summed, resulting in the total area of newly formed bone, being the value expressed in percentage of neoformed bone^25^,^26^.

#### Immunohistochemistry

Histological specimens (4-µm thick) were collected on silanized slides for better adhesion of the biological material studied and then kept in an oven for 24 hours at 37°C. After deparaffinization and hydration, histological sections were marked with a hydrophobic pen and then washed in a Tween enriched buffer solution twice for 3 minutes. Afterwards, the sections were immersed in a hydrogen peroxide for 10 minutes and then washed in phosphate buffer solution (PBS) twice in 3 minutes and finally immersed in inactivate endogenous peroxidase for 30 minutes.

The samples were separated in two groups, of which one was incubated with anti-Col-I (Santa Cruz Biotechnology, Dallas, TX, United States) polyclonal primary antibody at the concentration of 1:100, whereas the other was incubated with anti-Rank-L polyclonal primary antibody (Santa Cruz Biotechnology, Dallas, TX, United States) at the concentration of 1:200. Both groups were incubated for 2 hours and afterwards were washed twice in PBS. They were then submitted to a secondary antibody (anti-rabbit IgG) (Vector Laboratories, Burlingame, CA, United States) at the concentration of 1:200 in PBS for 30 min. After this process, the samples were washed in PBS three times before application of the avidin-biotin complex conjugated with peroxidase (Vector Laboratories, Burlingame, CA, United States) for 45 minutes. Visualization of the bound complexes was performed with application of 0.05% 3’3 diaminobenzidine solution, and contrast was given by Harris hematoxylin (Vector Laboratories, Burlingame, CA, United States)^27^.

The immunomarking of Collagen-I (Col-I) and the receptor of nuclear factor kappa-Β ligant (Rank-L) were qualitatively and semi-quantitatively evaluated. The qualitative analysis indicated the presence of a brownish immunostaining, and the semi-quantitative analysis was carried out by capturing three consecutive fields, using a light microscope (Leica Microsystems, Wetzlar, Germany). In the semi-quantitative analysis, the score 1-4 (1 = absent, 2 = mild, 3 = moderate, and 4 = severe) was used^28^,^29^. All analysis was performed by an experienced pathologist in a blind study.

### Statistical analysis

The data of means and standard deviations were submitted to normality tests, using the Shapiro-Wilk test for all variables. For the comparison of the experimental groups, the non-parametric Mann-Whitney test was used. Data were obtained through the SciPy library of Phyton 3 software, using the significance level of 5% (p ≤ 0.05).

## Results

### Histopathological analysis

Histopathological analysis revealed different tissue and structural morphological characteristics related to the experimental periods evaluated.

**Figure 2:**
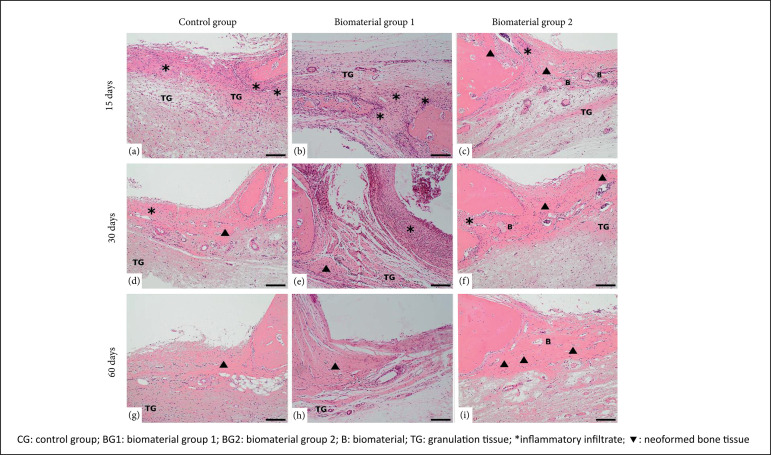
Photomicrographs representative of experimental groups. **(a)** Control group 15 days, **(b)** Biomaterial group 1 at 15 days, **(c)** Biomaterial group 2 at 15 days, **(d)** Control group at 30 days, **(e)** Biomaterial group 1 at 30 days,**(f)** Biomaterial group 2 at 30 days, **(g)** Control group at 60 days, **(h)** Biomaterial group 1 at 60 days, **(i)** biomaterial group 2 at 60 days. Coloration: hematoxylin and eosin (HE), bar = 40 µm, objective increase x10.

In the 15-day experimental period, moderate inflammatory infiltrate was observed in the CG with a slight presence of granulation tissue throughout the lesion, similar to that found in BG1, that presented moderate inflammatory infiltrate with presence of granulation tissue throughout the lesion (Figs. 2a-b). In contrast, BG2 demonstrated discrete particles of the biomaterial, a discrete inflammatory infiltrate with greater deposition of granulation tissue when compared to the other groups. It was also possible to observe evidence of the onset of bone formation in BG2 (Fig. 2c).

In the 30-day experimental period, CG presented mild inflammatory infiltrate, neoformed bone tissue with moderate amount of granulation tissue, similarly to that observed in BG1, but with biomaterial and neoformed bone tissue at the edges of the lesion. BG2 demonstrated the presence of the biomaterial, greater area of newly formed bone tissue when compared to CG and BG1 and greater amount of granulation tissue in the lesion area. In addition, it was possible to see in the BG2 trabecles interconnected through the presence of collagen fibers, which characterizes a greater support of the osteoblasts that aid the osteiointegration between biomaterial and tissue (Figs. 2d-f).

In the experimental period of 60 days, the CG presented new bone tissue with granulation tissue. BG1 presented particles of the biomaterial, slight presence of granulation tissue and greater area of neoformed bone tissue when compared to CG, as well as smaller area when compared to BG2 (Figs. 2g-i). BG2 also presented small particles of biomaterial, with a greater area of bone tissue neorformed when compared to CG and BG1 groups, and light presence of granulation tissue.

### Morphometric assessment

**Figure 3:**
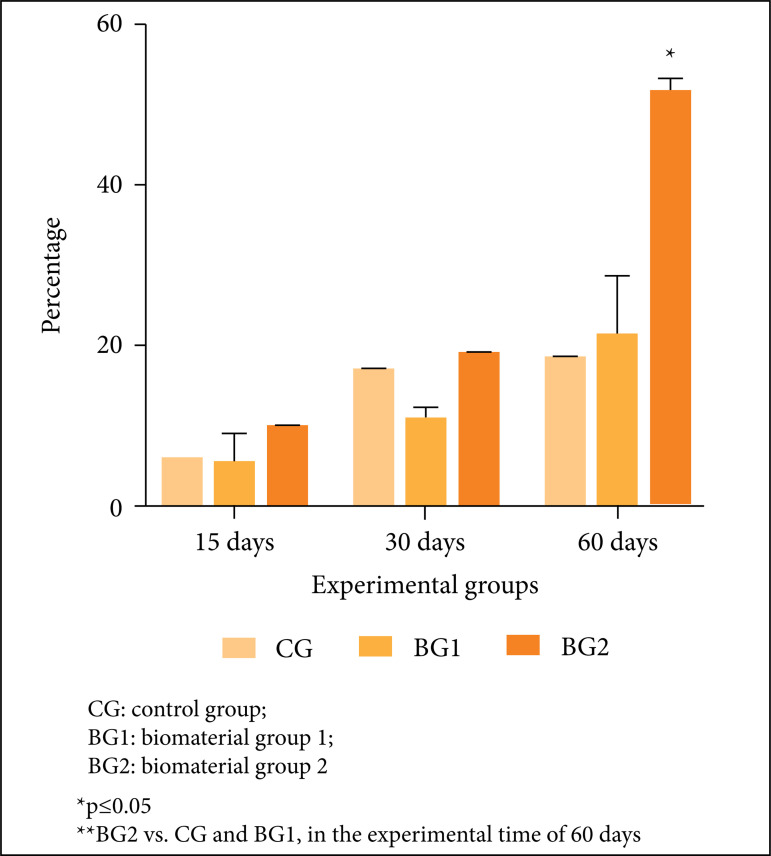
Representative graph of morphometric analysis **.

In the histomorphometric analysis, histological findings were confirmed, in which the treated groups presented a more advanced healing process, demonstrating greater bone formation when compared to the control. BG2-15presented 9.49% of neoformed bone compared to5.3% presented by CG-15 and 1.5% presented by BG1-15.BG2-30 presented 18.4% of neoformed bone compared to 17.5% presented by CG30 and 3.73% by BG1-30. GB2-60 presented the largest area of neoformed bone (50.9%), compared to 18.06% presented by CG60 and 7.06% by BG1-60. Although BG2 presented the largest area of newly formed bone in all experimental periods, the statistically significant difference was only found in the 60-day period when comparing BG2 with the other two groups. There was no statistically significant difference between CG and BG1 at any evaluated experimental time (Fig. 3).

### Immunohistochemistry

#### Collagen I

**Figure 4:**
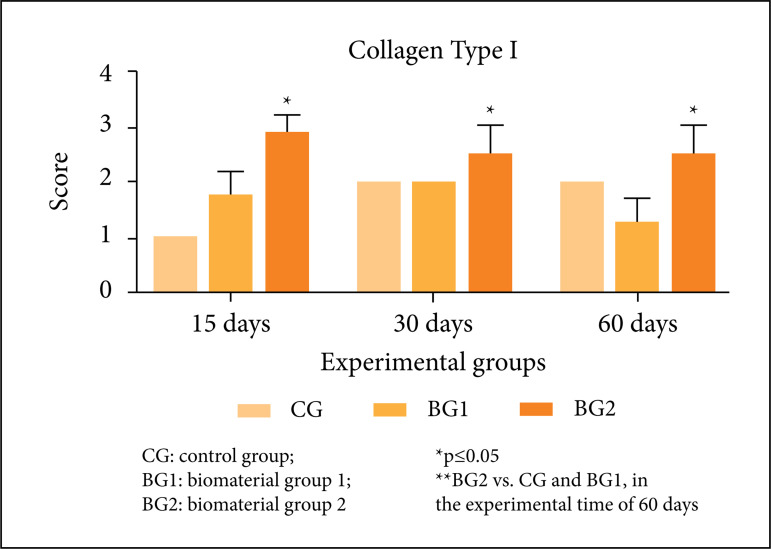
Representative graph of imunohistochemistry analysis of Collagen-I *,**.

Figure 4 represents the semi-quantitative analysis of the Col-I performed through score. The results showed significant statistical differences between the amount of collagen fibers present in each evaluated experimental group. BG2 presented the highest amount of fibers in all the experimental periods (15, 30 and 60 days) when compared to CG and BG1. In the comparison between CG and BG1 groups, no significant statistical differences were found.

#### Receptor activator of nuclear factor kappa-Β ligand

**Figure 5:**
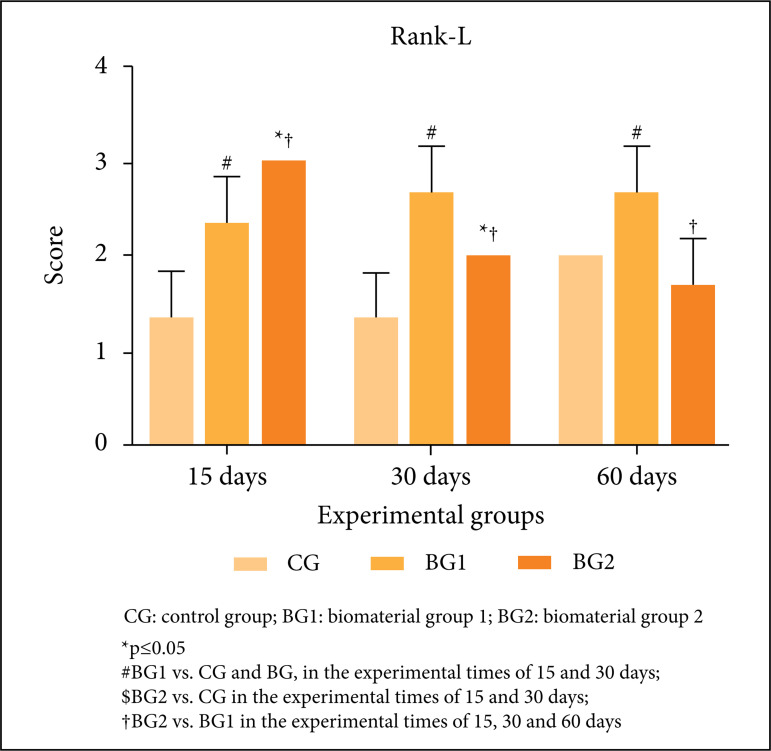
Representative graph of imunohistochemistry analysis of Rank-L*,

The semi-quantitative analysis of the Rank-L factor revealed differences between the analyzed groups. A statistically significant difference was found between BG2 and CG at 15 and 30 days, with BG2 showing the highest immunoexpression. In the period of 60 days, no difference was observed between the groups (BG2 and CG). In the comparison between BG1 and CG, the statistical difference was present in the experimental periods evaluated (15, 30 and 60 days), and BG1 presented the greater immunoexpression. When comparing the BG2 and BG1 groups, significant differences were also observed in all experimental periods evaluated. In the period of 15 days, BG1 presented less immunoexpression than BG2, and in the periods of 30 and 60 days BG1 presented greater immunoexpression than BG2 (Fig. 5).

## Discussion

The purpose of this study was to investigate the action of two different types of *scaffolds* based on the association of HA and PLGA on critical bone defects induced in calvaria of rats. Tissue engineering for bone grafts has been expanding in the last decades and has been considered as strategic therapy to minimize possible complications caused by critical defects.

The key component of this therapy are the *scaffolds*, as they support the formation of new bone tissue with structural characteristics that favors cellular interactions and the new extracellular matrix formation^30^. In addition, characteristics such as shape, size, porosity, rate of degradation and biological behavior are indispensable, since such differences act directly on the rate and time of bone reconstitution.

Studies have shown that the combination of HA and PLGA can reduce some adverse reactions, as well as increase the activity of osteoblasts^31^-^36^. Specifically in our study, we used a *scaffold* already known and reported in the literature composed of HA and PLGA basically, and we present a new *scaffold* formed with the same base, but adding a hemostatic component. Our results demonstrate that the new *scaffold* (HA/PLGA/Bleed) induced superior cellular responses to that found in the *scaffold* composed only by HA and PLGA and also to the control. Therefore, such results induce to consider that this fact may be related to the Bleed component addition.

It should be noted that the component with hemostatic properties and designated by DMC company as Bleed, when in contact with blood, has the ability to absorb blood plasma and form a kind of gel as in coagulation. Platelets, red blood cells and other blood constituents are concentrated on the surface of this gel, accelerating the process of hemostasis. In this way, the blood clot is absorbed more quickly and replaced by granulation tissue, with intense angiogenesis, proliferation of fibroblasts and endothelial cells. Frequently, it is established that, for adequate progression of the repair process, coagulation and hemostasis are fundamental. When these two phases occur with the appropriate quality, they optimize the time involved in the repair of critical defects, which would explain the fact that the group that received the *scaffold* with the compound Bleed presented the best evolution on repair process when compared to the other groups.

On the other hand, in the isolated evaluation of the groups, the results obtained by implementing *scaffolds* only with HA/PLGA suggest that the mechanical resistance promoted by HA when in combination with PLGA is adequate and compatible with bone tissue. However, its degradability occurs slowly, which causes its 3D structure to remain for a longer time in the cellular environment. As a consequence, the replacement of *scaffold* by the new bone matrix also occurs in a slower and gradual way, which could explain the difference found in the groups treated in our study, in which the HA/PLGA had less bone substitution and a greater presence of the biomaterial in the times analyzed.

In addition, the literature reports that HA has been widely used in bone *scaffolds* due to its conductive activity and it is frequently used, directly or in conjunction, with other materials in clinical practice. Modified HA particles may aid in stabilizing the mechanical properties of *scaffolds* composed of PLGA and thus improve conduction ability by increasing the calcium surface for osteoblast ossification. Simultaneously, the collagen matrix along with the autologous stem cells may improve the osteogenic activity^37^. The results of Zhang *et al*.^38^ demonstrate that *scaffolds* produced by tissue engineering and HA/PLGA compounds and cells can significantly increase bone repair and regeneration capacity. The results showed that the functionalization of this *scaffold* with cells facilitated cell adhesion and proliferation reaching biological effects was superior to what was found in *scaffolds* produced with PLGA alone.

Similarly to our study, Zhong *et al*.^39^ evaluated two different types of *scaffolds* in-vitro and in-vivo studies. The results found in the in-vitro study demonstrated that *scaffold* composed of nHA/PLA had better performance related to cell adhesion, deposition of the new bone matrix than those with only PLGA in its formulation. On the other hand, Tayton *et al*.^40^ evaluated *scaffolds* with different in-vitro and in-vivo compounds (PLA, PLA+10% HA, PLGA, PLGA+10% HA). The in-vitro results demonstrated that all polymers showed optimal biocompatibility, but PLA showed the highest osteoblastic activity, which was concluded in the in-vivo assays, in which PLA/HA *scaffolds* showed ideal osteoinductive and osteogenic capacity with increased local bone formation and excellent activity in the formation of new vessels.

Bone fracture repair is literally considered a regenerative process^41^. Physiologically, it is considered that the mechanism involved in bone repair is dynamic because it involves events such as coagulation, recruitment of pro-inflammatory cells, cell proliferation and synthesis of a new matrix, especially the synthesis of collagen^42^. Didactically, the bone repair process is divided into distinct phases that overlap in a given time, defined between 1-7 days for inflammation, 7-10 days for regeneration, and later the remodeling that follows with the formation of new tissue by the orchestrated participation between the action of osteoblasts and osteoclasts^43^. From the results obtained in this study, it was possible to observe that BG2 presented in the evaluated period an early inflammation resolution when compared to BG1 and the control, which favored the cell migration necessary for the formation of the structure of the new tissue matrix and the necessary local mineralization. Such findings are observed both by histology and by the increase in bone formations shown in the morphometry graph.

Analyzing the process phases more intrinsically, we observed that collagen is considered the main protein belonging to the extracellular and structural matrix ofthe tissues, being classified as type I the most abundant in thecomposition of the bone tissue. They are synthesized by osteoblasts in a rich matrix that coordinates the process of mineralization through an extremely regulated and not yet fully understood process^37^. However, there are indications that calcium crystals are deposited in an organized manner among the newly synthesized fibers, which contributes to the final mineralization result^37^,^44^. In our study, we observed that BG2 presented high immunoexpression when compared to the other groups evaluated. This fact suggests that the greater amount of fibers induced high tissue quality, with ideal structure and support that contributed to the rapid bone mineralization. It is possible to compare our findings with the studies by Pinheiro*et al*.^45^ and Attia *et al*.^46^, who used a *scaffold* composed of HA/?-TCP and micro-HA, respectively. They observed an increase in both the deposition of collagen fibers and HA, thus concluding that the biomaterial induced a response in the repair process.

Another interesting event to be observed in a remodeling assessment is the metabolic balance expressed between bone formation and absorption^47^. Therefore, this event requires a synchronized activity between osteoblasts and osteoclasts. Rank-L are cytokine critical participants of this process and responsible for the survival, expansion and in-vitro differentiation and participant of the mechanism of bone resorption^48^,^49^. Authors report their greater expression during the later stages of the repair process in which the bone resorption mechanisms, necessary for the finalization of the remodeling, occur^49^-^51^. In addition, Nogueira *et al*.^51^ reported that, when a marked immunoexpression of this factor occurs around the biomaterial, this is associated to the process of particle degradation in the lesion environment. This fact would favor the activity of the osteoblasts that will be concentrated in the restoration and replacement of the same for the formation of a new matrix.

Our findings referring to BG1 corroborate with the studies already mentioned, in which it was observed that the biomaterial formed by HA/PLGA presented higher expression of this factor at later times (30 and 60 days). This suggests that the mechanical resistance of this type of biomaterial causes its degradability to occur more slowly, and therefore may have induced a greater osteoclastogenic response in these periods, when the Rank-L factor was more concentrated around the biomaterial particles, thus assisting its degradation and possible replacement of the same by bone tissue.

Currently, the literature presents a diversity of *scaffolds* directed to the aid of the bone critical defect repair mechanism. A large expansion in the use of this type of lesion has expanded in recent years, due to the similarity in the morphofunctional aspects related to the evaluation of the evolution kinetics of the bone repair process. The HA/PLGA *scaffolds* have already been shown, presenting interesting results regarding adequate resistance and prolonged residence in the lesion environment, in which the mechanism of particle replacement by the new tissue becomes viable, but often time-consuming, as presented in our study.

On the other hand, the modification in the formulation of this same *scaffold*, that is, the addition of a natural product with hemostatic properties, favored the bone process in order to induce specific factors such as the formation of the new extracellular matrix with high mineralization, which extended from the edges of theme defect to its central region. It is important to emphasize that this study was the first to investigate the new *scaffold* developed and proposed by our research group. Therefore, future studies will be carried out in order to specifically investigate the cellular components involved in the cascade coagulation cascade and how much it contributes to the evolution of the other phases.

## Conclusions

The new *scaffold* had an interesting biotechnological potential, as it managed to induce specific morphological and biological responses that help the cellular connection necessary for the bone regeneration phase to occur. Thus, it is possible to consider that a positive response was obtained in the tissue environment with the inclusion of the bleed hemostatic agent, and the results found in this group were superior to those presented by the *scaffolds* only with HA/PLGA. However, future studies will still be needed in order to prove these benefits and to further explore the molecular mechanisms that are activated in the early stages of the repair process.
